# Mass Balance Studies of Robenidine Hydrochloride in the Body of Channel Catfish (*Ictalurus punctatus*)

**DOI:** 10.3390/ani13233745

**Published:** 2023-12-04

**Authors:** Lei Zhang, Xiangxuan Du, Xiaohui Ai, Yongtao Liu

**Affiliations:** 1 College of Fisheries and Life Science, Shanghai Ocean University, Shanghai 201306, China; zl521199@163.com (L.Z.); assisic@163.com (X.D.); 2 Yangtze River Fisheries Research Institute, Chinese Academy of Fishery Sciences, Wuhan 430223, China; 3 Hubei Province Engineering and Technology Research Center for Aquatic Product Quality and Safety, Wuhan 430223, China; 4 Key Laboratory of Control of Quality and Safety for Aquatic Products, Ministry of Agriculture, Beijing 100141, China

**Keywords:** robenidine hydrochloride, HPLC–MS/MS, channel catfish, mass balance, drug residue

## Abstract

**Simple Summary:**

Robenidine hydrochloride (ROBH) is a synthetic anticoccidial drug in global aquaculture. The abuse and inappropriate use of ROBH may result in mortality in animals and drug residues in foodborne animals. Mass balance studies not only provide insights into the drug disposal process in animals, but also provide information on the metabolite composition and elimination pathways, leading to the identification of pharmacological metabolites. However, there are no related reports of mass balance studies for ROBH in fish. In addition to prodrugs, ROBH may include PCBA and PCHA as major metabolites. Therefore, this study was conducted to monitor ROBH levels and possible metabolites in Channel catfish (*Ictalurus punctatus*) to provide a theoretical basis for the development of maximum residue limits (MRLs) for ROBH in aquatic animals.

**Abstract:**

This study aims to determine the mass balance of robenidine hydrochloride (ROBH) in the body of Channel catfish (*Ictalurus punctatus*). ROBH was administered orally at a dose of 20 mg/kg; following drug administration, the water samples were collected at predetermined time points (12, 24, 48, 72, 96, 120, 144, and 168 h), the experimental fish were executed after the water samples were obtained at 168 h, and the tissue samples were collected separately from the bones. The water and tissue samples were analyzed by high-performance liquid chromatography coupled with tandem mass spectrometry (HPLC–MS/MS) for concentrations of ROBH and its potential major metabolites, 4-chlorohippuric acid (PCHA) and 4-chlorobenzoic acid (PCBA). The tissue samples were prepared using a modified QuEChERS procedure; the water samples were prepared using a liquid–liquid extraction (LLE) procedure. The results show that the recovery rate of ROBH in fish is very low, less than 2% of the total amount of the drug, and the recovery in water can reach 80.7% of the total amount of the drug. The content of PCBA accounted for 42.4% of the total amount of the drug; the content of ROBH accounted for 38.3% of the total amount of the drug. The content of PCHA accounted for less than 1% of the total amount of the drug. The results show that, after a single administration, ROBH is rapidly metabolized in vivo and excreted in the form of ROBH as well as metabolite PCBA. ROBH and PCBA can be used as the main targets for the metabolism detection of ROBH in Channel catfish.

## 1. Introduction

Channel catfish (*Ictalurus punctatus*) is a North American catfish and has the highest production rate among freshwater-farmed fish in North America. It is the most preferred farmed fish species because of its rapid development cycle, edible nature, ease of capture, and excellent meat quality, which has a high nutritional value and can boost immunity and blood cholesterol levels. The need for the increased production of Channel catfish has resulted in the progressive expansion of its breeding areas and density, as well as a subsequent increase in fish diseases. Parasitic illness is one of the main reasons aquaculture suffers financial losses [1,2].

ROBH is a synthetic anticoccidial drug, which is a guanidinium derivative, interfering with the synthesis of adenosine triphosphate and altering the metabolic pathway of parasite proteins [3,4]. In 1970, Kantor first reported ROBH’s efficient and broad-spectrum anticoccidial effect on chickens [5]. In China, for the purpose of helping to prevent coccidiosis in chickens and rabbits, the Hubei Institute of Pharmaceutical Industry successfully produced ROBH for the first time in 1977 and jointly demonstrated the drug’s preventative and controlling effects on the animals. Since then, ROBH has been widely utilized as a medicated feed additive [6,7]. ROBH is also very effective in preventing carp coccidiosis, and feeding ROBH (200 mg/kg) to fish for 12 d ensures zero infection in carp [8]. At present, ROBH is still one of the three kinds of feed additives permitted by the Ministry of Agriculture and Rural Development (MARD) for the prevention of coccidiosis in chickens and rabbits, and it has been used in China for more than 40 years; the issue of drug resistance has become more of a concern. The FDA recommends ROBH levels in chickens to be 30 mg/kg. The Chinese Veterinary Pharmacopoeia recommends levels of 10–15 mg/kg in chickens and rabbits, and suggests that chickens with levels at 30 mg/kg for a long period of time will produce a stinky smell in chicken meat and eggs. The Chinese Veterinary Pharmacopoeia also recommends a dose of 20 mg/kg in fish. However, because the effective dose is so close to the safe dose, it cannot be used to exert the drug’s effects when using insufficient measurements, and overdosing will result in the deaths of farm animals [9,10,11].

At present, it is considered that the residue marker of ROBH is a target residue [12,13]. Liu [14] developed a method that used high-performance liquid chromatography (HPLC) coupled with HESI-MS/MS, which could simultaneously determine the ROBH content and its metabolites in fish; the paper suggested that ROBH as well as PCBA could be used as target residues of ROBH in fish. Tian’s study showed that the metabolism of ROBH in rabbits occurred mainly in the form of prodrugs excreted in the feces, with a small amount of prodrugs excreted in the urine. Tian [15,16] concluded that PCBA and PCHA may also be the main metabolites of ROBH due to the differences in the lifestyles of fish and mammals, and that it would be of interest to investigate the metabolism of ROBH in fish further [1,17].

Although ROBH has been approved for use in aquaculture, there are no specifications to limit its maximum residue limits (MRLs) in aquatic products. To develop an MRL for ROBH in fish, it is important to clarify its uptake, distribution, and metabolite recovery rates in excreta and tissue. Mass balance studies not only provide insights into the drug disposition process in animals, but also provide information on metabolite composition and elimination pathways, which can lead to the identification of pharmacological metabolites [18,19]. However, the mass balance studies of ROBH in fish have not been reported yet. Though investigations have revealed that PCBA and PCHA may also be the substance’s primary metabolites, ROBH is presently thought to be the residual marker [20,21]. Therefore, this study chose China’s main farming species, the Channel catfish, as the research object to conduct a mass balance study of ROBH, aiming to provide a theoretical basis for the development of the MRLs of ROBH in fish bodies.

## 2. Materials and Methods

### 2.1. Experimental Animals

The experimental fish (*n* = 24; body mass: 50 ± 10 g) were provided by the experimental base of the Yangtze River Fisheries Research Institute of the Chinese Academy of Fisheries Sciences. The fish were acclimatized to laboratory conditions in the aquarium for 1 week before use in the experiments. During acclimatization, the fish were fed with 1% BW full-price feed but not treated with antibacterial drugs. The test water was tap water, which was aerated continuously for 48 h to keep the dissolved oxygen at greater than 5.0 mg/L. The pH and temperature of the test water were 7.5–8.0 and 25 °C, respectively.

### 2.2. Reagents

Robenidine Hydrochloride (Purity ≥ 98%, Quality ratio, Dr. Ehrenstorfer GmbH, Augsburg, Germany), Robenidine-D8 Standard (Purity ≥ 98%, Quality ratio, Dr. Ehrenstorfer GmbH, Augsburg, Germany), and Robenidine Hydrochloride Raw Material (CAS 25875-50-7, Purity 98%, Shouguang Huachih Chemical Co., Shandong, China) were used. Ethyl acetate, hexane, and formic acid (chromatographic purity, J.T. Baker Co., NJ, USA). Anhydrous ethanol (analytically pure, Xilong Chemical Co., Ltd., Fujian, China), distilled water, soluble starch (analytically pure, Xiangzhong Chemical Reagent Supply Station, Shanghai, China), anhydrous magnesium sulfate (analytically pure, Sinopharm Chemical Reagent Co., Ltd., Shanghai, China), and heparin sodium (biological grade, Shanghai Biochemical Reagent Co., Ltd., Shanghai, China). Mettler-TOLE-DOAE-240 precision electronic balance (Mettler-Toledo, Zurich, Switzerland), 20PR-520 high-speed refrigerated centrifuge (Hitachi, Japan), speed mixer (Shanghai Kanghua, Shanghai, China), nitrogen blower (AOSHENG, Hangzhou Aosheng Instrument Co., Ltd., Zhejiang, China), rotary evaporator (Gongyi Yuhua, Henan, China), HPLC–Tandem Mass Spectrometer (Surveyor MS Pump Plus, Surveyor Autosampler Plus, Thermo TSQ Quantum Access MAX and Thermo LCquan 2.6 data acquisition system, Thermo Fisher Scientific, MA, USA).

### 2.3. Drug Treatment and Sample Collection

Material balance experiment: 9 tails of experimental fish were placed in 9 small glass tanks (20 cm × 30 cm × 30 cm), forming 7 experimental groups and 2 blank groups, with each group comprising 1 tail of experimental fish. Each tank contained 4 L of water, and before the experiment started, the experimental fish were restrained for two days. The experimental groups were administered ROBH orally at a dose of 20 mg/kg and body weights were recorded. The water samples were collected at predetermined time points (12 h, 24 h, 48 h, 72 h, 96 h, 120 h, 144 h, and 168 h), and the experimental fish were executed after the water samples were taken at 168 h. Blood was collected with 2 mL injector, which was then rinsed by 1% heparin sodium; muscle, skin, kidney, intestine, liver, gill, eye, and brain were collected with scissors. All the tissues were thoroughly stirred and mixed. Each time, a 0.5 L water sample was collected after the water was mixed thoroughly. After the collection was complete, the water in which the experimental fish lived was completely replaced with a clean equal volume of water. Then, the mixed tissue and water samples were stored at −20 °C for testing.

Under the same light conditions and the same temperature conditions, the dosage given to 50 g of experimental fish was splashed directly into a small glass tank. A total 50 mL water sample was collected from each group at 12 h, 24 h, 48 h, 72 h, 96 h, 120 h, 144 h, and 168 h after the completion of the spraying, and the water samples were stirred thoroughly before collection.

### 2.4. Sample Preparation

For water sample analysis, the water samples were thawed at room temperature. Water tissue samples were prepared with the following LLE procedure: 20 mL of water sample was transferred into a 50 mL graduated polypropylene centrifuge tube; then, 400 μL of IS solution (1 mg/L) was added and the solution was vortex-mixed for 30 s. After that, the sample was allowed to stand for 10 min, and 15 mL of ethyl acetate containing 1% formic acid was added and shaken for 30 s. Next, 0.8 g of Na_2_SO_4_ was added to the sample, and then vortexed for 30 s. The sample was left to stand to separate the organic phase, and the organic phase was taken out in the heart-shaped bottle. The residues were re-extracted with 15 mL of ethyl acetate containing 1% formic acid. The organic phases from the two separations were combined into the same heart-shaped bottle and concentrated to 2 mL in a rotary evaporator at 45 °C. The heart-shaped bottle was washed with an extra 5 mL of ethyl acetate containing 1% formic acid to prevent residue on the bottle, and the organic phase was transferred into a 10 mL graduated polypropylene centrifuge tube. The organic phase was dried under a gentle stream of nitrogen at 45 °C. The residue was dissolved with 1 mL of 1% formic acid and methanol (72:28, *v*/*v*) and vortexed for 30 s. The obtained solution was centrifuged at 10,000 rpm for 5 min and filtered through a 0.22 μm filter.

For mixed tissue analysis, the homogenized samples were thawed at room temperature. Mixed tissue samples were prepared with the following modified QuEChERS procedure: 1 g of sample was transferred into a 15 mL graduated polypropylene centrifuge tube; 20 μL of IS solution (1 mg/L) was added, then vortex-mixed for 30 s. After that, the sample was allowed to stand for 10 min, and 5 mL of ethyl acetate containing 1% formic acid was added. Then, the sample was shaken vigorously for 30 s and placed in an ultrasonic bath for 5 min. Next, 1 g of MgSO_4_ was added to the sample, which was then vortexed for 30 s and centrifuged at 8000 rpm for 5 min. The extract was collected into another 15-mL graduated polypropylene centrifuge tube. The residues were re-extracted with 3 mL of acetonitrile containing 1% formic acid and the extraction procedure without adding MgSO_4_ described above was carried out. The consecutive extracts were placed into the same graduated polypropylene centrifuge tube and dried under a gentle stream of nitrogen at 45 °C. The residue was reconstituted with 1 mL of 1% formic acid and methanol (72:28, *v*/*v*) and 1 mL hexane, then vortex-mixed for 30 s. The obtained solution was centrifuged at 10,000 rpm for 5 min and the hexane was removed, using 1 mL of 1% formic acid with methanol (72:28, *v*/*v*) filtered through a 0.22 μm filter.

### 2.5. Instrument Conditions

Column: Symmetry^®^ C18 (100 mm × 2.1 mm × 3.5 μm); flow rate: 300 μL/min; injection volume: 20.0 μL; column temperature: 30 °C.

Mass spectrometry conditions: heated electrospray ionization source (HESI), positive and negative ions in simultaneous scanning mode, ROBH and ROBH-d_8_ were scanned in positive mode, PCBA and PCHA were scanned in negative mode, detection was performed in selected reaction monitoring (SRM) mode; spray voltage: 3000 V; sheath gas pressure: 40 kPa; auxiliary gas pressure: 5 kPa; evaporating gas temperature: 350 °C; collision gas and pressure: argon, 1.5 MPa; ion transfer capillary temperature: 350 °C. The precursor ion of ROBH is 344.07 m/z; the products ions are 154.94 m/z (CE, 22 eV) and 137.96 m/z (CE, 26 eV); and the IS is ROBH-d_8_, for which the precursor ion is 342 m/z and the products ion is 159 m/z (CE, 21 eV) in positive mode. The precursor ion of PCBA is 154.94 m/z, and the products ions are 110.96 m/z (CE, 16 eV) and 113.27 m/z (CE, 12 eV). The precursor ion of PCHA is 212.01 m/z, and the products ions are 167.98 m/z (CE, 14 eV) and 110.91 m/z (CE, 18 eV). PCBA and PCHA were both scanned in negative ion mode.

### 2.6. Preparation of Standard Curves and Determination of Recovery and Precision

Standard stock solutions were prepared by dissolving 10 mg ROBH, PCBA, PCHA, and ROBH-d8 standards in methanol and diluted to a final concentration of 100 mg/L, respectively. Then, 1 mL ROBH, PCBA, PCHA, and ROBH-d8 standard stock solutions were taken; diluted to 10 mL with 1% formic acid and methanol (72:28, *v*/*v*), respectively; and a single standard solution with a mass concentration of 10 mg/L was prepared. After that, six concentrations of ROBH, PCBA, and PCHA standard solutions (10, 20, 50, 100, 200, and 500 μg/L) were diluted, which were contained in screw thread amber glass bottles and stored at −20 °C.

Six concentrations of ROBH, PCBA, and PCHA standard solutions (10, 20, 50, 100, 200, and 500) and 20 μg/L IS were added into blank samples and prepared under the method in 2.4, then analyzed using the method in 2.5. Standard calibration curves for ROBH, PCBA, and PCHA were constructed by the ratio of plotting peak areas and the peak area of IS versus six concentrations (10, 20, 50, 100, 200, and 500 µg/kg) of ROBH, PCBA, and PCHA in 1% formic acid and methanol (72:28, *v*/*v*), respectively.

In order to calculate recoveries, samples spiked at three different levels (20, 100, and 200 µg/L) were analyzed in five blank replicas. The peak areas of the analytes recovered from the samples were compared with those of the target chemicals in the standard solutions.

### 2.7. Data Processing

The standard curve was plotted using Microsoft Excel 2013 software.

## 3. Results and Analyses

### 3.1. Applicability of Testing Methods

**Figure 1:**
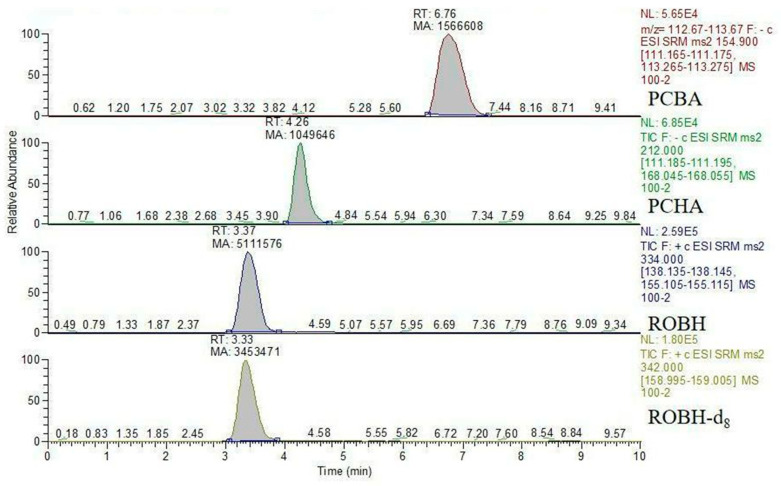
Chromatograms of 100 µg/kg ROBH, PCBA, and PCHA standards spiked in a Channel catfish (*Ictalurus punctatus*) water sample. The peak times were approximately 6.76 min for PCBA, 4.26 min for PCHA, 3.37 min for ROBH, and 3.33 min for ROBH-d_8_.

The limits of quantification (LOQ) for ROBH, PCBA, and PCHA in water and tissues were 5 μg/kg, and the limit of detection (LOD) was 10 μg/kg (Figure 1). Matrix-matched standard solution curves were obtained for ROBH, PCBA, and PCHA in this experiment as follows: y = 0.0012x − 0.0067 (R^2^ = 0.9997), y = 4440.7x − 16056 (R^2^ = 0.9998), and y = 9994.7x + 11764 (R^2^ = 0.9999). The recovery rates in the water of ROBH were 77–123%, the recovery rates in the water of PCBA were 74–115%, the recovery rates in the water of PCHA were 96–107%, and their relative standard deviations (RSDs) were all less than 7%. The recovery rates in tissues of ROBH were 76–129%; the recovery rates of PCBA in tissues were 75–98%; the recovery rates of PCHA in tissues were 70–101%; and the RSDs were all less than 10%, which indicates that the method is suitable for the detection of ROBH, PCBA, and PCHA in water as well as in tissues (Table 1).

### 3.2. The Rate of Dissipation of ROBH in Water

**Figure 2:**
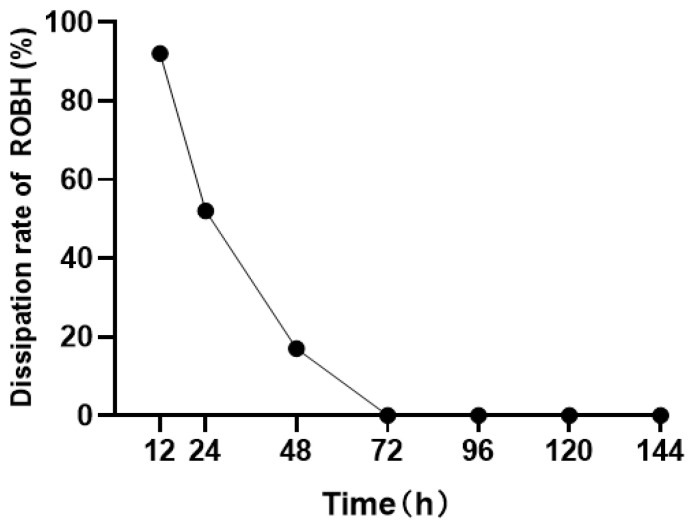
Dissipation rates of ROBH in water.

Under the same light and temperature conditions, the rate of dissipation is shown in Figure 2. The dissipation rate decreased to 92% after 12 h, 52% after 24 h, and 17% after 48 h. No other metabolites were detected. Therefore, the pre-experiment proved that in the experimental group, it is necessary to replace all the water at the end of each sampling to ensure that the detection target will not degrade too much, as it may cause too many mistakes.

### 3.3. Recovery of ROBH and Its Metabolites In Vitro and In Vivo in the Channel Catfish

**Figure 3:**
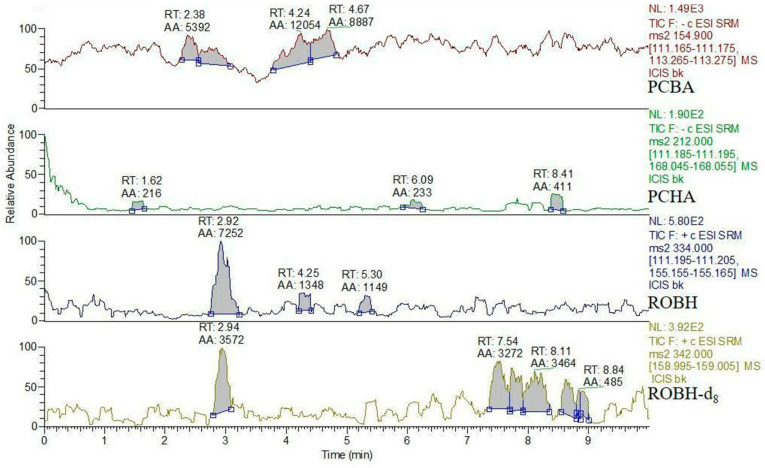
Chromatograms of blank water in which the fish survived during the experiment. The peak times were approximately 6.76 min for PCBA, 4.26 min for PCHA, 3.37 min for ROBH, and 3.33 min for ROBH-d_8_.

**Figure 4:**
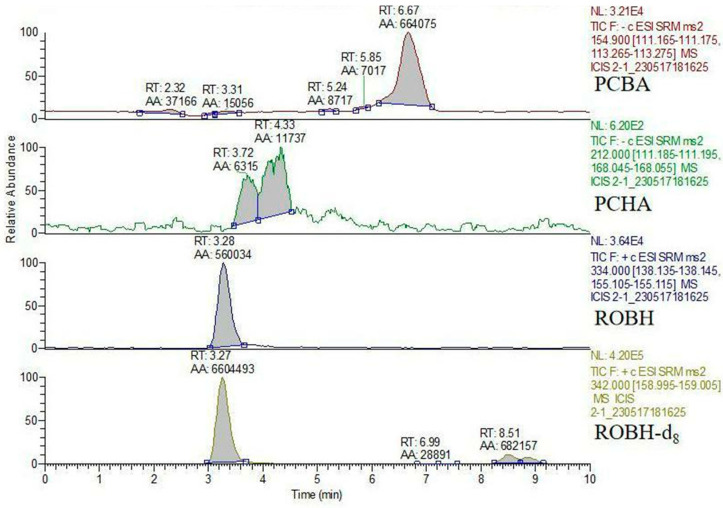
Chromatograms of ROBH and its metabolites detected in water samples obtained between 12 and 24 h after a single oral instillation of ROBH. The peak times were approximately 6.76 min for PCBA, 4.26 min for PCHA, 3.37 min for ROBH, and 3.33 min for ROBH-d_8_.

**Figure 5:**
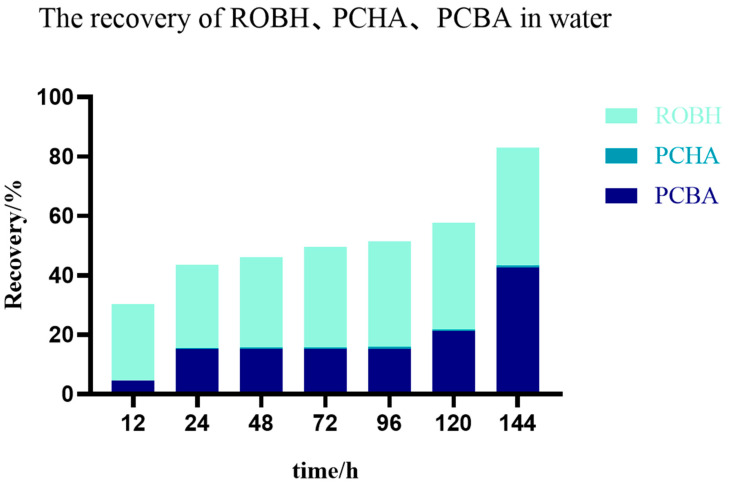
Enrichment of ROBH and PCBA excreted into water after a single oral administration at room temperature and under the same light condition.

In this study, ROBH, PCBA, and PCHA were not detected in the blank experimental group (Figure 3), while the targets were detected in the water samples of the experimental group; so, it is obvious that ROBH, PCBA, and PCHA were all detected (Figure 4) [22]. After collecting the water body on the seventh day of a single dose, the experimental fish were culled of their bones and blood, and the liver, kidney, gill, and intestine were collected and mixed. An insignificant quantity of drug residues could be found in the mixed tissue, the levels of ROBH and PCBA were below 10 μg/kg, and PCHA was not detected. The recovery of ROBH in the environmental water at 12, 24, 48, 72, 96, 120, and 144 h can be found in Table 2. In the water, the content of PCBA accounted for 42.41% of the total amount of drug, the content of ROBH accounted for 38.25% of the total amount of drug, the content of PCHA accounted for less than 1% of the total amount of drug, and the content of the drug detected in the water accounted for 82% of the total amount of drug after 144 h. The total amount of the drug detected in the water after mixing was 82% (Figure 5).

## 4. Discussion

### 4.1. Selection of Extraction Methods

For the extraction of water bodies, solid-phase extraction and the LLE procedure can both be used. Pre-experimentation of blank water samples with standard working solution added using a solid-phase extraction method revealed that the precision test results did not fit the standard and the recovery was also too low; so, the LLE method was used.

Referring to the relevant literature, methanol, acetonitrile, and ethyl acetate were examined as extraction solvents, and it was found that the extraction effect of ethyl acetate was better in urine. Robenidine is composed of a guanidine moiety. Guanidine derivatives have resonance-stabilized cations and are strong bases, due to the conjugated acid of the guanidinium group having a pKa of around 13.5 and staying protonated over a broad pH range. Consequently, it was calculated that the acid treatment assisted in keeping certain analytes, including basic robenidine, in their neutral forms in addition to freeing bound residues from the matrix [1,15,23]. Examining the extraction effect of hydrochloric acid, acetic acid, and other acidified ethyl acetates, the two extraction recoveries are comparable, taking into account the volatility of hydrochloric acid, and the final choice is a volume fraction of 0.1% formic acid acidified ethyl acetate as a solvent for the extraction of ROBH in the water body.

### 4.2. Metabolic Pattern of ROBH in the Channel Catfish

The principal metabolites of ROBH in the Channel catfish in this work were ROBH and PCBA. PCHA was also clearly discovered in the experiment, but its concentration was too low to be regarded as the primary metabolite. In rabbits, ROBH was quickly assimilated and eliminated; 80% of it was recovered in feces and 20% in urine. The oxidation of tetrachlorobenzaldehyde to tetrachlorobenzoic acid is the first step in the metabolic pathway of ROBH in rabbits, which is followed by conjugation with glycine to form PCBA species [24]. The final step in the metabolic process is the hydrolysis of the semidihydrazine carbonyl link. The three main metabolites of ROBH contribute to less than 10% of the excretion in feces, with the prodrug form accounting for 70% to 80%. In this experiment, feeding was interrupted during the experiment and Channel catfish’s fecal excretion was reduced, as it had to metabolize the drug by way of excretion through bodily fluids, like rabbits’ urine. In the dissipation experiment of ROBH, under the same light and temperature, only ROBH was detected in the water. In the blank experimental group, none of these three chemicals were detected, indicating that the experimental group of PCBA was totally detected in the fish metabolism. Consequently, PCBA can be used as a major metabolite in the body of Channel catfish.

### 4.3. Mass Balance Studies of ROBH in the Channel Catfish

In comparison to the earlier study, the pharmacokinetics of ROBH in the Channel catfish body demonstrated that following the metabolism of ROBH in the body of Channel catfish, detection of the content of the drug vanished after 120 h [25]; it was also difficult to detect the drug in the mix samples collected 168h after a single dose in this study. This indicates that ROBH and PCBA were essentially fully discharged into the water within 168 hours. Regarding drug detection in the water column, the content of PCBA accounted for 42.41% of the total drug, the content of ROBH accounted for 38.25% of the total drug, and the content of PCHA accounted for less than 1% of the entirety of drug. At 144 h, the drug detected in the water accounted for 80.66% of the entire drug. Of the total ROBH excretion, 50% was excreted in the first 12 h, which was 16.3% of the total drug content. The remaining excretion of ROBH occurred during the following 144 h and was progressively excreted. In the first 24 h, PCBA, which was 16% of the total drug content, was excreted; the content of the excretion was reduced between 24 h and 96 h; and it started to increase after 96 h. The excretion of PCBA from 96 h to 144 h was responsible for 36.9% of the total recovery and 32% of the total drug content. In the pharmacokinetic experiments, ROBH was present in a variety of matrices and was excreted from the body in large amounts in the 12–24 h and 96–144 h periods. All matrices, except the muscle and the liver, reached their peak levels of ROBH within these time frames. In this study, one peak appeared at 12 h, and another appeared at 24 h and 96 h, which was consistent with the experiment’s findings. Following the ROBH in the body of Channel catfish, PCBA levels in the water body started to rise after 96 h [14]. It is speculated that it may be because PCBA is a hydrolysis product of ROBH, the excretion pathway of fish is different from that of other animals, and there is a large amount of water in the environment; so, PCBA becomes the highest content of excreta in the aqueous environment.

### 4.4. Recovery of ROBH in Animals

The excretion recovery test of drugs is an important aspect of the study of the metabolic process of drugs in vivo. By determining the content of drugs and their metabolites in different excretions, it is possible to verify the entry of drugs into the circulation and their main metabolic pathways. After exogenous drugs are administered through internal administration, part of them are absorbed into the body circulation through the small intestine and other parts of the body, while the others are excreted through the intestines along with feces [22,26]. The elimination of drugs entering circulation generally takes place in two ways: one is that they are directly excreted as the original drug without any metabolic transformation, and the other is that they are metabolized into other metabolites in the liver and other tissues by metabolic enzyme catalysis and then excreted through the bile or kidneys [25,27].

Zulalian’s study on the metabolism of ROBH in rats by 14C labeling showed that ROBH was finally excreted in the feces as 70.4% of the total dose; 2.0% of the total dose of robenidine was excreted in urine as PCHA and PCBA, which were the main metabolites, accounting for 12% of the total radioactivity, with PCBA accounting for 98%. Zulalian’s study of ROBH in chickens in C-labeled form showed that 99% of the total radioactivity was present in the feces and that most of this radioactivity was in the form of robenidine prodrugs, while some was in the form of metabolites.

## 5. Conclusions

This study determines the excretion pattern of ROBH in the body of Channel catfish and presents an accurate, stable, and reliable HPLC–MS/MS with a method of scanning positive and negative ions together for simultaneous determination of ROBH, PCHA, and PCBA in fish samples and water samples. To the best of our knowledge, this study is the first report on the determination of both ROBH and its metabolites in fish and its water environment simultaneously. A complete verification of the method was conducted, covering accuracy and recovery. Results showed good accuracy, stability, and low LOQs. ROBH excreted into the environment accounted for 38.25% of the total amount of oral instillation, and the content of PCBA accounted for 42.41% of the total amount of oral instillation. This indicates that ROBH and PCBA are the main metabolites excreted from the body of Channel catfish. These results may be used as reference data for the safety and responsible use of ROBH in fish.

## Figures and Tables

**Table 1:** The method validation of ROBH, PCBA, and PCHA in water and tissues (n = 5).

Chemicals	Spiked Level (μg/kg or μg/L)	Water			Tissues		
		Recovery Rate (%)	Intra-Day RSD (%)	Inter-Day RSD (%)	Recovery Rate (%)	Intra-Day RSD (%)	Inter-day RSD (%)
ROBH	20	77–123	1.4	3.42	95–129	3.1	0.61
	100	82–120	1.1	6.21	76–110	3.4	0.74
	200	99–101	1.6	5.10	87–116	1.6	6.54
PCBA	20	74–115	1.7	6.81	80–97	3	7.68
	100	90–100	1.0	3.50	77–95	0.5	4.99
	200	97–101	0.8	4.32	75–98	0.5	3.3
PCHA	20	104–107	1.4	6.81	81–101	0.5	9.83
	100	96–103	1.7	3.93	70–99	2.5	0.48
	200	96–100	1.6	6.86	72–95	0.8	8.53

**Table 2:** The recovery of ROBH in the environmental water at 12, 24, 48, 72, 96, 120, and 144 h.

Dose	Number	Time (h)								Total Amount of Recycling (µg)	Recovery (%)	Total Recovery (%)
		Chemicals	12	24	48	72	96	120	144			
1160	1	PCBA	33.2	243.4	7.1	<LOD	<LOD	19.7	157.5	460.7	39.72	76.88
		PCHA	<LOD	<LOD	<LOD	<LOD	<LOD	<LOD	<LOD	0	0	
		ROBH	236.8	24.9	23.3	24	24	24	74	431	37.16	
1200	2	PCBA	57	87.2	<LOD	22.8	10.2	67.8	238.9	482.9	40.24	79.82
		PCHA	<LOD	<LOD	<LOD	<LOD	<LOD	<LOD	<LOD	0	0	
		ROBH	304	23	25	33.3	<LOD	35.7	46	475	39.58	
800	3	PCBA	52.2	33.5	12.9	15.9	<LOD	22.1	231.8	332.4	4.16	80.65
		PCHA	<LOD	<LOD	<LOD	<LOD	<LOD	<LOD	<LOD	0	0	
		ROBH	129.1	144.2	163	163.4	222.5	18.2	72	312.7	39.09	
1360	4	PCBA	70.7	183.9	14.7	<LOD	<LOD	47.9	59.1	570.9	41.98	81.38
		PCHA	<LOD	<LOD	<LOD	<LOD	<LOD	<LOD	<LOD	0	0	
		ROBH	214	168	72.8	<LOD	49.2	34	8.9	535.9	39.40	
1000	5	PCBA	24.3	184.8	<LOD	41.2	10.9	27.2	218.4	502.8	50.28	83.75
		PCHA	<LOD	<LOD	<LOD	<LOD	<LOD	<LOD	<LOD	0	0	
		ROBH	155.6	48.1	10.8	24.1	10.6	40.2	45.3	334.7	33.47	
800	6	PCBA	44.5	79.6	45.3	<LOD	17.8	43.4	131.1	361.7	45.21	83.69
		PCHA	<LOD	<LOD	<LOD	<LOD	<LOD	<LOD	<LOD	0	0	
		ROBH	130	25	15	23	25	39	60.8	307.8	38.48	
1120	7	PCBA	55.3	125.1	<LOD	13.3	11.1	1.9	217.8	424.5	37.90	78.5
		PCHA	<LOD	<LOD	<LOD	<LOD	<LOD	<LOD	<LOD	0	0	
		ROBH	225.2	125.5	24.3	14.1	12.3	<LOD	53.6	454.7	40.60	

## Data Availability

The datasets used and analyzed during the current study are available from the corresponding author on reasonable request.
