# Correction: Zhang et al. Mass Balance Studies of Robenidine Hydrochloride in the Body of Channel Catfish (*Ictalurus punctatus*). *Animals* 2023, *13*, 3745

**DOI:** 10.3390/ani14060971

**Published:** 2024-03-21

**Authors:** Lei Zhang, Xiangxuan Du, Xiaohui Ai, Yongtao Liu

**Affiliations:** 1 College of Fisheries and Life Science, Shanghai Ocean University, Shanghai 201306, China; zl521199@163.com (L.Z.); assisic@163.com (X.D.); 2 Yangtze River Fisheries Research Institute, Chinese Academy of Fishery Sciences, Wuhan 430223, China; 3 Hubei Province Engineering and Technology Research Center for Aquatic Product Quality and Safety, Wuhan 430223, China; 4 Key Laboratory of Control of Quality and Safety for Aquatic Products, Ministry of Agriculture, Beijing 100141, China

## Text Correction

There were errors in the original publication [1]. We miswrote “Chlorpheniramine”; this is a phonetic word, and the official word exists.

A correction has been made to the title, Simple Summary, Abstract, Keywords, Section 2.2. Reagents, Section 4.4. Recovery of ROBH in Animals, and Reference 6.

The title has been rewritten as “Mass Balance Studies of Robenidine Hydrochloride in the Body of Channel Catfish (*Ictalurus punctatus*)”.

In the Simple Summary, “Chlorpheniramine hydrochloride (ROBH) is a synthetic anticoccidial drug in global aquaculture”. has been rewritten as “Robenidine hydrochloride (ROBH) is a synthetic anticoccidial drug in global aquaculture”.

In the Abstract, “This study aims to determine the mass balance of chlorpheniramine hydrochloride (ROBH) in the body of Channel catfish (*Ictalurus punctatus*)”. has been rewritten as “This study aims to determine the mass balance of robenidine hydrochloride (ROBH) in the body of Channel catfish (*Ictalurus punctatus*)”.

In the Keywords, “Chlorpheniramine Hydrochloride” has been rewritten as “Robenidine hydrochloride”.

In Section 2.2. Reagents, Section 4.4. Recovery of ROBH in Animals, and Reference 6, “chlorpheniramine” has been rewritten as “Robenidine”.

The authors state that the scientific conclusions are unaffected. This correction was approved by the Academic Editor. The original publication has also been updated.
